# Compassion in Balance: A Cross‐Sectional Study on Satisfaction and Fatigue Among Clinical Nurses at HMC

**DOI:** 10.1002/hsr2.72183

**Published:** 2026-04-08

**Authors:** Surekha Kiran Patil, Rajesh Rai, Badriya Abdulla Al‐Lenjawi, Kiran Patil, Kalpana Singh

**Affiliations:** ^1^ Hamad Medical Corporation Doha Qatar; ^2^ D Y Patil University Deemed to be University Mumbai Maharashtra India; ^3^ Surg Art Hospital Doha Qatar

**Keywords:** burnout, clinical nurses, compassion fatigue, compassion satisfaction, Hamad Medical Corporation, professional quality of life, Qatar, secondary traumatic stress

## Abstract

**Background and Aim:**

Nursing is a profoundly rewarding yet emotionally demanding profession. Clinical nurses often experience a delicate balance between compassion satisfaction (CS) and compassion fatigue (CF), which can significantly impact their well‐being and patient care. The study aims to investigate the levels of compassion satisfaction and compassion fatigue among clinical nurses at Hamad Medical Corporation (HMC) and to find out the association between various demographic characteristics and these outcomes.

**Methodology:**

A cross‐sectional study was conducted among 219 clinical nurses at HMC, Doha, Qatar, between May 2023 and September 2023. Convenience sampling was used. Data was collected using a demographic survey and the Professional Quality of Life Scale 5 (ProQOL 5). Data analysis was conducted using STATA 17.0, applying both descriptive and inferential statistical methods. Associations between the variables were explored using one‐way ANOVA and Pearson correlation coefficient tests.

**Results:**

Among the 219 participants, the majority reported average levels of Compassion Satisfaction (CS) (*n* = 205; 93.6%), Burnout (BO) (*n* = 209; 95.4%), and Secondary Traumatic Stress (STS) (*n* = 167; 76.3%). Statistically significant associations were found between compassion satisfaction and nationality (*p* < 0.001), job designation (*p* = 0.006), and burnout and job designation (*p* = 0.007). Additionally, a moderate positive correlation was identified between burnout and secondary traumatic stress (*r* = 0.5249, *p* < 0.001).

**Conclusion:**

The majority of nurses reported average levels of compassion satisfaction, burnout, and secondary traumatic stress, with significant associations observed across selected demographic and professional variables and a moderate positive correlation between burnout and secondary traumatic stress. These findings emphasize the value of professional quality of life assessment in guiding evidence‐informed strategies to support nurse well‐being and sustain quality patient care.

## 1 Introduction

Nursing requires significant emotional involvement and a high level of empathy, which can lead to a delicate balance between experiencing compassion satisfaction and compassion fatigue [1]. While striving to provide quality care, nurses encounter both the rewarding and challenging aspects of their profession. Compassion satisfaction represents the positive emotional benefits and fulfillment that come from helping others, whereas compassion fatigue reflects the negative consequences, including burnout and secondary traumatic stress. This fatigue can lead to negative emotions toward their work and result in both physical and mental exhaustion [2, 3].

A study by Stamm utilizing the Professional Quality of Life (ProQOL) framework conceptualizes professional well‐being as the dynamic interaction between compassion satisfaction (CS) and compassion fatigue (CF), the latter comprising burnout (BO) and secondary traumatic stress (STS). The cumulative effects of these positive and negative caregiving experiences are encapsulated within the concept of professional quality of life (ProQOL). According to Stamm [4], achieving and maintaining an appropriate balance between compassion satisfaction and compassion fatigue is crucial for preserving nurses' emotional well‐being, professional functioning, and the quality of patient care.

Compassion fatigue is comprised of two key components: burnout and secondary traumatic stress. Burnout is characterized by physical and emotional exhaustion, a depletion of energy, and feelings of helplessness or hopelessness [5], secondary traumatic stress, another facet of compassion fatigue, often emerges in nurses who form deep empathetic connections with their patients. This intense empathy can lead nurses to internalize their patients' suffering, resulting in emotional distress. Compassion fatigue does not involve physical harm, it arises from the significant emotional burden associated with caring for others [6].

When compassion fatigue persists, it can impair a nurse's ability to remain objective, leading to flawed professional judgments, erroneous perceptions of patients' conditions, and a decline in the quality of care provided [7, 8]. Over time, this erosion of objectivity and the associated dread can compromise patient safety [2, 9]. In contrast, compassion satisfaction—the positive emotional reward derived from helping others—acts as a vital counterbalance to compassion fatigue. Unlike burnout, which typically stems from job dissatisfaction and can occur in any profession, secondary traumatic stress is specifically linked to caregiving roles and direct patient interactions. Thus, emphasizing the need to nurture compassion satisfaction [10].

The American Nurses Association [11] revealed that 82% of nurses feel they are at significant risk of illness due to workplace stress. Younger nurses, who make up 40% of the nursing workforce, are particularly vulnerable to burnout, stress, and compassion fatigue. These challenges are further intensified by rapid technological advancements, which place additional demands on nurses and threaten their overall well‐being. Compounding these issues is the global nursing shortage, with a projected shortfall of 7.6 million nurses by 2030 [12]. The high expectations placed on nurses further compound their emotional burden, increasing the risk of fatigue and negatively affecting patient outcomes.

Moreover, the emotional and psychological toll of nursing is compounded by the high expectations placed on these professionals to deliver exceptional care under often challenging conditions. The combination of long hours, high patients load, and the emotional burden of caregiving can create a perfect storm for compassion fatigue, further threatening the well‐being of nurses and the safety of the patients they care for [3, 5, 9, 13].

Fostering compassion satisfaction is therefore not only crucial for individual well‐being but also a strategic priority for healthcare organizations aiming to maintain high standards of patient care. The Qatar National Health Strategy [14] underscores the importance of enhancing the health and well‐being of healthcare staff as a key component in achieving the broader goals of Qatar Vision 2030, which aspires to create a world‐class healthcare system that meets the needs of its population.

This study aims to investigate the levels of compassion fatigue and compassion satisfaction among clinical nurses at Hamad Medical Corporation (HMC) and was to find the association between demographic variables. By identifying the key determinants of compassion fatigue and satisfaction, this research seeks to inform the development of targeted interventions that can support the well‐being of nurses, align with the objectives of Qatar's national health priorities, and ultimately contribute to the delivery of high‐quality patient care.

### 1.1 Hypotheses

The following hypotheses were formulated to guide the study:

Compassion satisfaction among clinical nurses is significantly associated with selected demographic variables, including age, nationality, designation, years of experience, and academic qualification.

Burnout and secondary traumatic stress among clinical nurses are significantly associated with selected demographic variables.

Burnout is positively correlated with secondary traumatic stress, while compassion satisfaction is inversely related to components of compassion fatigue.

## 2 Methods and Materials

### 2.1 Study Design and Setting

A quantitative cross‐sectional design was employed for this study, targeting a total of 219 clinical nurses across three key facilities: Hamad General Hospital (HGH), the Surgical Specialty Center (SSC), and the Medical Care and Research Center (MCRC). These facilities operate under the umbrella of Hamad Medical Corporation (HMC), Doha, Qatar. HMC serves as the principal public healthcare sector in the State of Qatar and is widely recognized for delivering specialized, high‐quality tertiary healthcare services across the nation.

### 2.2 Sampling Technique, Sampling Frame, and Selection Criteria

The sampling frame comprised licensed registered nurses working in inpatient units at HGH, SSC, and MCRC during the study period. Email invitations, together with study information, were distributed through institutional mailing lists to approximately 1000 nurses across the three facilities.

The sample size was calculated using STATA version 17.0, based on a 95% confidence level and a 5% margin of error, yielding a required sample of 219 participants. A convenience sampling technique was utilized to recruit participants.

**Figure 1:**
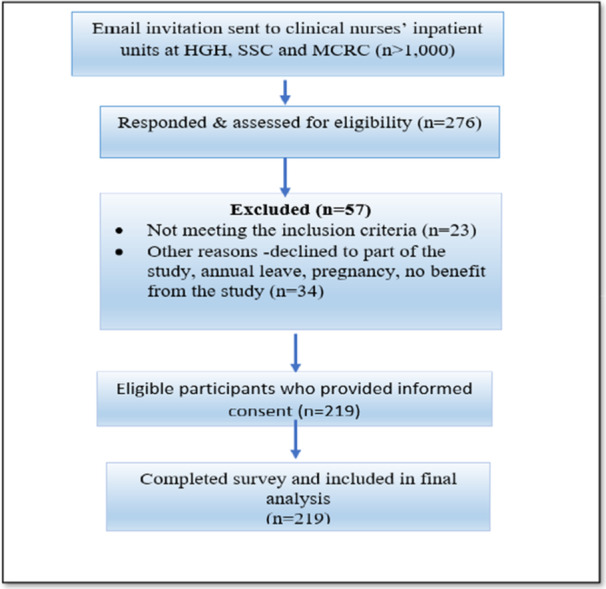
Sampling frame and participant selection flow diagram.

A total of 276 nurses responded to the invitation. Eligibility was assessed using predefined inclusion and exclusion criteria during the screening process. Of these respondents, 23 did not meet the inclusion criteria, and 34 declined participation or were unavailable (e.g., due to leave or pregnancy, or perceived lack of benefit of the study.). Consequently, 219 eligible nurses provided informed consent and completed the survey, resulting in a 79.3% response rate among eligible respondents. The recruitment and selection process is illustrated in Figure 1.

### 2.3 Inclusion and Exclusion Criteria

The inclusion criteria encompassed licensed registered clinical nurses working in inpatient areas and involved in direct patient care. Nurses who were newly hired and under preceptorship, those assigned to outpatient departments, while new staff under preceptorship, outpatient department nurses, and those unwilling to participate were excluded.

### 2.4 Data Collection Tools

Data collection involved the use of a structured questionnaire comprising two main components. The first section gathered demographic information, including gender, age in years, nationality, academic qualification, years of experience in HMC, area of work, marital status, and self‐care practices.

The second section utilized the Professional Quality of Life Scale 5 (ProQOL 5), a validated self‐report instrument designed to measure both the positive and negative effects of working in caregiving professions. The tool evaluates CS and CF, with CF conceptualized as a construct comprising BO and STS, as defined by Stamm [4]. The tool consists of 30 items distributed across three subscales: CS, BO, and STS. Each item is rated on a 5‐point Likert scale ranging from 1 (“never”) to 5 (“very often”). Subscale scores are calculated by summing the ratings for the relevant items. Higher scores on the CS subscale reflect greater professional satisfaction, whereas higher scores on the BO and STS subscale indicate increased levels of burnout and secondary traumatic stress, respectively. ProQOL 5 has demonstrated strong psychometric properties, with subscale Cronbach's alpha coefficients ranging from 0.84 to 0.90, and an overall reliability coefficient of 0.91, confirming its suitability for assessing the well‐being of healthcare professionals.

### 2.5 Data Collection

Data collection was conducted between May 2023 and September 2023 following approval from the relevant institutional authorities. Eligible nurses were invited through the organization's official email system. The principal investigator or a research team member provided a research information sheet and explained the study objectives, procedures, duration, and voluntary nature of participation. Participants were given 2 weeks to consider participation, with follow‐up reminder emails sent to enhance recruitment.

Participants who expressed willingness were screened for eligibility using a predefined checklist. Those meeting the inclusion criteria were enrolled in the study. Participation was voluntary and anonymous, and written informed consent was obtained prior to data collection. Participants then completed the demographic questionnaire and the ProQOL‐5, which required approximately 20–25 min to complete.

### 2.6 Ethical Approval

This study was conducted in full conformance with principles of the “Belmont Report.” The Permission letter was obtained from the Institutional Review Board (MRC‐01‐22‐581) of HMC before data collection. The research was executed in compliance with HMC and MoPH human participant research ethics rules and regulations.

### 2.7 Data Analysis

All statistical analyses were performed using the statistical packages STATA version 17.0 (StataCorp LLC, College Station, TX, USA) and Epi Info version 7.2, (Centers for Disease Control and Prevention, Atlanta, GA, USA). STATA is a powerful statistical analysis and data visualization tool used in different fields by researchers. Descriptive and inferential statistical measures were used to analyze the data. Furthermore, the data analyzed was summarized and presented in appropriate tables. For the association one‐way Analysis of Variance (ANOVA) and the Pearson correlation coefficient test was used. A *p* value of less than 0.05 was considered statistically significant, and all tests were conducted using a two‐tailed approach.

## 3 Results

### 3.1 Demographic Characteristics

The questionnaire was answered by 219 participants. All participants completed the ProQOL 5 tool. Table 1 presents the distribution of demographic characteristics of the participants. The sample included 184 (84%) females. diverse range of nationalities, with the largest groups being Indian 133 (60.7%) and Filipino 74 (33.8%). The age distribution shows that most nurses are in their 30 s, with 152 (69.4%) of participants falling within the 31–40‐year age range, indicating a relatively young workforce.

**Table 1:** Demographic characteristics of the participants (*n* = 219).

Variables	*F*	%	Variables	*F*	%
Gender			Designation		
Male	35	16.0%	Staff nurse	201	91.8%
Female	184	84.0%	Charge nurse	18	8.2%
Nationality			Age in years		
Indian	133	60.7%	26–30	25	11.4%
Filipino	74	33.8%	31–40	152	69.4%
Arab/other	12	5.5%	41–50	29	13.2%
			51–60	13	5.0%
Area of work			Year of experience in HMC		
Medical	46	21.0%	Less than 1 year	8	3.7%
Surgical	30	13.7%	1–5 years	92	42.0%
Critical care	86	39.3%	6–10 years	53	24.2%
Emergency	36	16.4%	10–15 years	32	14.6%
Pediatric	21	9.6%	> 15 years	34	15.5%
Academic qualification			Marital status		
Diploma	13	5.9%	Married	189	86.3%
BSN	203	92.7%	Unmarried	29	13.2%
MSN	3	1.4%	Divorce	1	0.5%
Previous self‐care practices					
Yes	122	55.70%			
No	97	44.30%			

Mostly the nurses hold the designation of staff nurse 201 (91.8%), holds BSN degree 203 (92.7%) are working across various units, with a significant concentration in critical care 86 (39.3%), followed by medical 46 (21%) and surgical 30 (13.7%) units. Regarding marital status data indicates that the majority of participants are married 189 (86.3%). Further, in terms of experience, most nurses have between 1 and 5 years of experience at HMC 92 (42%), although there is a notable representation of more experienced nurses with 6–10 years 53 (24.2%). Regarding self‐care practices, 55.70% practiced and 44.30% had not practiced self‐care previously, with common practices including self‐talk, relaxation, and gratitude exercises.

### 3.2 Compassion Satisfaction and Compassion Fatigue of Study Participants

The levels of CS, BO, and STS were evaluated using standardized scoring guidelines, where scores < 22 indicate low, 23–41 indicate average, and ≥ 42 indicate high levels [4]. Among the participants, the majority (*n *= 205; 93.6%) reported average CS, while a smaller proportion experienced low CS (*n* = 4; 1.8%) or high CS (*n *= 10; 4.6%). Regarding BO, most participants (*n *= 209; 95.4%) had average levels, with only 4 (1.8%) and 6 (2.7%) reporting low and high levels, respectively. In terms of STS, low levels were reported by 51 participants (23.3%), while 167 participants (76.3%) indicated average levels, and only one participant (0.5%) experienced high STS. These findings suggest that the majority of clinical nurses reported moderate levels of both CS and CF (Table 2).

**Table 2:** Compassion satisfaction and compassion fatigue of the study participants.

Levels	Low (< 22) *n* (%)	Average (23–41) *n* (%)	High (≥ 42) *n* (%)
Compassion satisfaction level	4 (1.8%)	205 (93.6%)	10 (4.6%)
Burnout level	4 (1.8%)	209 (95.4%)	6 (2.7%)
Secondary traumatic stress level	51 (23.3%)	167 (76.3%)	1 (0.5%)

### 3.3 Mean Difference Compassion Satisfaction and Compassion Fatigue

Mean compassion satisfaction scores were broadly comparable across most sociodemographic and work‐related variables, with overall values clustering around the mid‐30s. The only statistically significant difference was observed by nationality, where Indian nurses reported a mean CS score of 33.9 ± 5.3, Filipino nurses 34.4 ± 4.0, and Arab/other nationalities 33.5 ± 6.2 (*p* = 0.018), corresponding to a small‐to‐moderate effect size (partial *η*² = 0.04). No statistically significant differences in CS were identified by gender (male: 34.4 ± 6.1 vs. female: 33.9 ± 4.8; *p* = 0.59; *d* = 0.09), age group (*p* = 0.68; *η*² = 0.02), designation (*p* = 0.86; *d* = 0.04), clinical area (*p* = 0.54; *η*² = 0.02), years in the current unit (*p* = 0.12; *η*² = 0.03), academic qualification (*p* = 0.55; *η*² = 0.02), or marital status (*p* = 0.66; *d *= 0.08), with all effect sizes indicating negligible to small practical differences (Table 3).

**Table 3:** Mean difference in compassion satisfaction and compassion fatigue.

Variable	Categories (*N*)	CS mean (SD)	CS *p* value	CS effect size	BO mean (SD)	BO *p* value	BO effect size	STS mean (SD)	STS *p* value	STS effect size
Gender	Male (35)	34.4 (6.1)	0.59	*d *= 0.09	31.7 (4.8)	0.53	*d* = 0.10	26.1 (5.4)	0.59	*d* = 0.11
	Female (184)	33.9 (4.8)			31.3 (3.9)			26.7 (5.8)		
Nationality	Indian (133)	33.9 (5.3)	0.018	*η*²*p* = 0.04	31.2 (4.4)	0.8	*η*²*p* = 0.01	27.0 (6.0)	0.31	*η*²*p* = 0.01
	Filipino (74)	34.4 (4.0)			31.7 (3.5)			25.7 (5.1)		
	Arab/others (12)	33.5 (6.2)			31.3 (3.6)			27.8 (6.6)		
Age (years)	26–30 (25)	32.9 (4.9)	0.68	*η*²*p* = 0.02	31.5 (4.7)	0.26	*η*²*p* = 0.02	25.7 (5.4)	0.051	*η*²*p* = 0.04
	31–40 (152)	34.2 (5.3)			31.4 (4.0)			26.2 (5.5)		
	41–50 (29)	33.8 (4.2)			31.9 (3.7)			29.3 (6.5)		
	51–60 (13)	34.2 (2.5)			29.2 (3.7)			26.0 (5.0)		
Designation	Staff nurse (201)	34.0 (4.9)	0.86	*d* = 0.04	31.5 (4.0)	0.088	*d* = 0.36	26.6 (5.6)	0.89	*d* = 0.03
	Charge nurse (18)	33.8 (5.6)			29.8 (4.9)			26.4 (6.7)		
Area of work	Medical (46)	33.1 (4.7)	0.54	*η*²*p* = 0.02	30.7 (4.4)	0.19	*η*²*p* = 0.03	25.5 (6.3)	0.36	*η*²*p* = 0.02
	Surgical (30)	34.0 (4.7)			32.8 (4.3)			27.9 (7.2)		
	Critical care (86)	34.3 (5.7)			31.3 (4.1)			26.4 (5.1)		
	Emergency (36)	33.8 (4.1)			31.6 (3.7)			27.4 (5.2)		
	Pediatric (21)	35.2 (3.9)			30.7 (3.1)			26.1 (4.9)		
Year of experience in HMC	< 1 year (8)	29.6 (8.4)	0.12	*η*²*p* = 0.03	29.1 (3.0)	0.57	*η*²*p* = 0.01	24.1 (4.6)	0.26	*η*²*p* = 0.02
	1–5 years (92)	34.2 (4.8)			31.5 (4.0)			26.1 (5.6)		
	6–10 years (53)	33.8 (4.7)			31.4 (3.4)			26.3 (4.9)		
	≥ 10 years (66)	34.4 (4.2)			31.2 (4.4)			27.8 (6.4)		
Academic qualification	Diploma (13)	35.4 (3.5)	0.55	*η*²*p* = 0.02	31.7 (4.3)	0.66	*η*²*p* = 0.01	30.2 (6.1)	0.059	*η*²*p* = 0.04
	BSN (203)	33.9 (5.1)			31.3 (4.0)			26.3 (5.6)		
	MSN (3)	33.0 (5.6)			33.3 (6.4)			27.7 (3.1)		
Marital status	Married (189)	34.0 (5.0)	0.66	*d* = 0.08	31.3 (4.2)	0.42	*d* = 0.15	26.7 (5.7)	0.3	*d* = 0.19
	Unmarried/divorced (30)	33.6 (4.8)			31.9 (3.2)			25.6 (5.4)		

*Note:* Values are presented as mean (SD). Effect sizes are reported as Cohen's *d* for *t*‐tests and partial eta squared (*η*²*p*) for ANOVA. A *p* value < 0.05 was considered statistically significant.

Abbreviations: BO = burnout, CS = compassion satisfaction, STS = secondary traumatic stress.

Burnout scores were relatively consistent across subgroups, with mean values ranging from approximately 29–33. No statistically significant associations were observed between burnout and nationality (Indian: 31.2 ± 4.4; Filipino: 31.7 ± 3.5; Arab/others: 31.3 ± 3.6; *p* = 0.80; *η*² = 0.01), gender (*p* = 0.53; *d* = 0.10), age (*p* = 0.26; *η*² = 0.02), designation (*p* = 0.088; *d* = 0.36), clinical area (*p* = 0.19; *η*² = 0.03), years in the unit (*p* = 0.57; *η*² = 0.01), academic qualification (*p* = 0.66; *η*² = 0.01), or marital status (*p* = 0.42; *d *= 0.15) (Table 3).

Secondary traumatic stress scores showed modest variability across participant characteristics, with mean values generally between 25 and 29. No statistically significant differences in STS were observed by nationality (Indian: 27.0 ± 6.0; Filipino: 25.7 ± 5.1; Arab/others: 27.8 ± 6.6; *p* = 0.31; *η*² = 0.01), gender (*p* = 0.59; *d* = 0.11), designation (*p* = 0.89; *d* = 0.03), clinical area (*p* = 0.36; *η*² = 0.02), years in the current unit (*p* = 0.26; *η*² = 0.02), academic qualification (*p* = 0.059; *η*² = 0.04), or marital status (*p* = 0.30; *d* = 0.19). Although STS scores were numerically higher in the 41–50‐year age group (29.3 ± 6.5), the overall age‐group comparison did not reach statistical significance (*p *= 0.051), and effect sizes remained small (Table 3).

### 3.4 Association of Demographic Variables With Levels of Outcome Variables

The analysis examined the relationship between demographic characteristics (gender, age, nationality, designation, area of work, years of experience, and qualifications) and the levels of compassion satisfaction and compassion fatigue (Table 4). Among nationalities, Indian nurses had the highest proportion in the high compassion satisfaction group (80.0%), while Filipino nurses were predominantly in the average group (35.6%) and showed no representation in the low category. Other Arab nationalities had small sample sizes but still contributed to variation. Regarding designation, all nurses with high compassion satisfaction were staff nurses (100%), while none of the charge nurses fell into the high category, and low scorers (50%) were charge nurses. Other demographic variables including gender, age area of work, years of experience in HMC, and academic qualification did not show statistically significant differences in compassion satisfaction levels.

**Table 4:** Association between demographic variables and compassion satisfaction levels.

Demographic variables	Compassion satisfaction level					
	Categories	*N*	Low	Average	High	
			*n* (%)	*n* (%)	*n* (%)	
			4	205	10	*p* value
Gender	Male	35	1 (25.0%)	31 (15.1%)	3 (30.0%)	0.40
	Females	184	3 (75.0%)	174 (84.9%)	7 (70.0%)	
Nationality	Indian	133	3 (75.0%)	122 (59.5%)	8 (80.0%)	**< 0.001**
	Filipino	74	0 (0.0%)	73 (35.6%)	1 (10.0%)	
	Arab/others	12	1 (25.0%)	10 (4.9%)	1 (10.0%)	
Age in years	26–30 years	25	0 (0.0%)	25 (12.2%)	0 (0.0%)	0.62
	31–40 years	152	4 (100%)	139 (67.8%)	9 (90.0%)	
	41–50 years	29	0 (0.0%)	28 (13.7%)	1 (10.0%)	
	51–60 years	13	0 (0.0%)	13 (6.3%)	0 (0.0%)	
Designation	Staff nurse	201	2 (50.0%)	189 (92.2%)	10 (100%)	**0.006**
	Charge nurse	18	2 (50.0%)	16 (7.8%)	0 (0.0%)	
Area of work	Medical	8	0 (0.0%)	44 (21.5%)	2 (20.0%)	0.34
	Surgical	92	1 (25.0%)	29 (14.1%)	0 (0.0%)	
	Critical care	53	3 (75.0%)	76 (37.1%)	7 (70.0%)	
	Emergency	32	0 (0.0%)	35 (17.1%)	1 (10.0%)	
	Pediatric	34	0 (0.0%)	21 (10.2%)	0 (0.0%)	
Years of experience in HMC	< 1 year	8	1 (25.0%)	6 (2.9%)	1 (10.0%)	0.12
	1–5 years	92	1 (25.0%)	88 (42.9%)	3 (30.0%)	
	6–10 years	53	0 (0.0%)	50 (24.4%)	3 (30.0%)	
	10–15 years	32	2 (50.0%)	28 (13.7%)	2 (20.0%)	
	> 15 years	34	0 (0.0%)	33 (16.1%)	1 (10.0%)	
Qualification	Diploma	13	0 (0.0%)	12 (5.9%)	1 (10.0%)	0.94
	BSN	203	4 (100.0%)	190 (92.7%)	9 (90.0%)	
	MSN	3	0 (0.0%)	3 (1.5%)	0 (0.0%)	

*Note:* Test statistics—*χ* ^2^ test and Fisher exact test. Bold values indicate *p* < 0.05 considered as significant.

The results indicated a statistically significant association between compassion satisfaction levels and nationality (*p* < 0.001) as well as designation (*p* = 0.006). Additionally, burnout levels were found to be significantly associated with designation (*p* = 0.007), while STS levels were significantly related to academic qualifications (*p* = 0.001). In contrast, other variables such as gender, age, area of work, and years of experience did not show any statistically significant associations with compassion satisfaction, burnout, or STS levels.

The analysis of burnout and STS levels across demographic variables reveals that designation is significantly associated with burnout levels, while academic qualification shows a significant association with STS levels (*p *= 0.001). Specifically, all nurses in the high burnout group were staff nurses (100%), and none were charge nurses, though charge nurses accounted for 50% of the low burnout group. This indicates that staff nurses are more likely to experience high burnout compared to charge nurses. For STS, although only one participant was in the high STS category, this individual had a qualification diploma, making the association statistically significant (*p *= 0.001). In contrast, no other demographic variables such as gender, nationality, age, area of work, years of experience, or qualification level showed significant associations with either burnout or STS levels (*p* values all > 0.05) (Table 5).

**Table 5:** Association between demographic variables and levels of compassion fatigue.

Demographic variables	*N*	Levels of burnout			*p* value	Levels of STS			*p* value
		Low	Average	High		Low	Average	High	
		*n* (%)	*n* (%)	*n* (%)		*n* (%)	*n* (%)	*n* (%)	
		4	209	6		51	167	1	
Gender									
Male	35	1 (25%)	33 (15.8%)	1(16.7%)	0.88	8 (15.7%)	27 (16.2%)	0 (0%)	0.91
Females	184	3 (75%)	176 (84.2%)	5(83.3%)		43(84.3%)	140(83.8%)	1(100%)	
Nationality									
Indian	133	3 (75%)	125 (59.8%)	5(83.3%)	1.00	29(56.9%)	103(61.7%)	1(100%)	0.95
Filipino	74	1 (25%)	72 (34.4%)	1(16.7%)		19(37.3%)	55 (32.9%)	0 (0%)	
Arab/others	12	0 (0%)	12 (5.8%)	0 (0%)		3 (5.8%)	9 (5.4%)	0 (0%)	
Age in years									
26–30 years	25	0 (0%)	23 (11%)	2(33.3%)	0.34	6 (11.8%)	19 (11.4%)	0 (0%)	0.26
31–40 years	152	3 (75%)	146 (69.9%)	3 (50%)		38(74.5%)	114(68.3%)	0 (0%)	
41–50 years	29	0 (0%)	28 (13.4%)	1(16.7%)		5 (9.8%)	23 (13.8%)	1(100%)	
51–60 years	13	1 (25%)	12 (5.7%)	0 (0%)		2 (3.9%)	11 (6.6%)	0 (0%)	
Designation									
Staff nurse	201	2 (50%)	193 (92.3%)	6 (100%)	**0.007**	46(90.2%)	154(92.2%)	1(100%)	0.86
Charge nurse	18	2 (50%)	16 (7.7%)	0 (0%)		5 (9.8%)	13 (7.8%)	0 (0%)	
Area of the department working									
Medical	8	0 (0%)	8 (3.8%)	0 (0%)	0.84	15(29.4%)	31 (18.6%)	0 (0%)	0.17
Surgical	92	2 (50%)	88 (42.1%)	2(33.3%)		6 (11.8%)	23 (13.8%)	1(100%)	
Critical care	53	0 (0%)	52 (24.9%)	1(16.7%)		22 43.1%)	64 (38.3%)	0 (0%)	
Emergency	32	1 (25%)	28 (13.4%)	3 (50%)		5 (9.8%)	31 (18.6%)	0 (0%)	
Pediatric	34	1 (25%)	33 (15.8%)	0 (0%)		3 (5.9%)	18 (10.8%)	0 (0%)	
Years of experience in HMC									
< 1 year	8	2 (50%)	43 (20.6%)	1(16.7%)	0.38	2 (3.9%)	6 (3.6%)	0 (0%)	0.63
1–5 years	92	1 (25%)	28 (13.4%)	1(16.7%)		23(45.1%)	69 (41.3%)	0 (0%)	
6–10 years	53	1 (25%)	82(39.2%)	3 (50%)		12(23.5%)	41 (24.6%)	0 (0%)	
10–15 years	32	0 (0%)	35 (16.7%)	1(16.7%)		7 (13.7%)	24 (14.4%)	1(100%)	
> 15 years	34	0 (0%)	21 (10%)	0 (0%)		7 (13.7%)	27 (16.2%)	0 (0%)	
Academic qualification									
Diploma	13	0 (0%)	12 (5.7%)	1(16.7%)	0.80	1 (2.0%)	11 (6.6%)	1(100%)	**0.001**
BSN	203	4(100%)	194 (92.8%)	5 83.3%)		50(98.0%)	153 (91.6%)	0 (0%)	
MSN	3	0(0.0%)	3(1.4%)	0(0.0%)		0(0%)	3(1.8%)	0(0%)	

*Note:* Test statistics—*χ* ^2^ test and Fisher exact test. Bold values indicate *p* < 0.05 considered as significant.

### 3.5 Correlation Between Outcome Variables

3.5

Pearson's correlation analysis was performed to evaluate the relationships between compassion satisfaction, burnout, and secondary traumatic stress among clinical nurses. The analysis revealed no significant correlation between compassion satisfaction and burnout (*r *= 0.035, *p*= 0.6064) or between compassion satisfaction and secondary traumatic stress (*r *= −0.0394, *p*= 0.5621). However, a significant positive correlation was found between burnout and secondary traumatic stress (*r *= 0.5249, *p* < 0.001), suggesting that higher levels of burnout are associated with higher levels of secondary traumatic stress (see Table 6).

**Table 6:** Correlation between outcome variables.

Pearson correlation	Compassion satisfaction and burnout	Compassion Satisfaction and Secondary traumatic stress	Burnout and secondary traumatic stress
*r* value	0.035	−0.0394	0.5249
*p* value	0.6064	0.5621	< **0.001**

*Note:* Test statistics—Pearson correlation. Bold values indicate *p* < 0.05 considered as significant.

## 4 Discussion

The findings of this study highlight the delicate balance between compassion satisfaction and compassion fatigue, underscoring the need for a comprehensive approach to supporting the well‐being of clinical nurses. The findings indicate that most clinical nurses experienced average compassion satisfaction alongside low to average levels of burnout and STS. These outcomes are consistent with previous research by Lee et al. [15], in which most nurses reported average scores across all ProQOL subscales. Similarly, Zhang et al. [16] likewise observed that average compassion satisfaction reflects a balanced professional fulfillment, though nurses with lower compassion satisfaction and higher burnout or STS may require targeted psychological support.

Analysis of demographic variables revealed notable trends in compassion satisfaction, burnout, and STS among clinical nurses. Although age‐related differences in STS were not statistically significant, nurses aged 41–50 demonstrated higher mean STS scores, suggesting potential mid‐career vulnerability, consistent with reports by Potter et al. [17]. Similarly, nurses with less than 1 year of experience showed lower ProQOL scores across all dimensions, possibly reflecting limited exposure and emotional engagement. While these trends were not statistically significant, they mirror findings by [18] and Pappa et al. [13], who identified younger and less experienced nurses as being at increased risk of burnout. Kelly et al. [7] and Kestler et al. [19] also reported higher compassion fatigue among early‐career nurses.

Staff nurses demonstrated higher burnout compared to charge nurses, supporting prior evidence that frontline roles carry a greater emotional burden. Nurses with more than 10 years of experience showed more stable ProQOL scores, suggesting that clinical maturity may enhance emotional resilience. Additionally, surgical nurses reported higher burnout and STS than nurses in other units, consistent with Rajeswari et al. [20], who highlighted the emotional strain associated with high‐intensity clinical environments. Nurses with diploma‐level education demonstrated significantly higher STS than those with advanced qualifications, reinforcing Beck's [21] assertion that educational preparation may buffer psychological distress.

Compassion satisfaction showed significant associations with nationality (*p* < 0.001) and job designation (*p* = 0.006). These associations are reported descriptively however, these findings should be interpreted cautiously due to subgroup imbalance and the cross‐sectional design, these results do not imply stable or generalizable differences but rather indicate patterns that warrant While Stamm [4] and Sacco et al. [22] similarly reported contextual influences on compassion satisfaction, the present study did not explore organizational, cultural or psychosocial mechanisms underlying these differences. Future research incorporating qualitative methods, organizational variables, and longitudinal designs is recommended to better understand the nature and stability of these associations.

Burnout was significantly associated with job designation (*p* = 0.007), consistent with earlier studies linking frontline care to emotional exhaustion due to high workloads and direct patient care responsibilities. STS was significantly related to academic qualification (*p* = 0.001), supporting evidence that educational level influences awareness and processing of traumatic exposure [21]. No significant associations were observed between gender, age, work area, or experience and ProQOL subscales, consistent with mixed findings reported by Hooper et al. [23] and Kelly et al. [7].

Pearson correlation revealed a strong positive relationship between burnout and STS (*p* < 0.001), indicating that these two dimensions of compassion fatigue are closely interrelated. This finding is consistent with Bahari et al. [24] and Rajan et al. [25], confirming the interconnected nature of compassion fatigue dimensions. In contrast, compassion satisfaction was not significantly correlated with burnout or STS, suggesting distinct underlying mechanisms [22].

Collectively, these findings reinforce existing literature advocating targeted interventions—such as resilience training, stress management, trauma‐informed care, and professional support systems—to reduce burnout and STS, while strategies including recognition, reflective practice, and peer support may enhance compassion satisfaction. These approaches align with recommendations by Craigie et al. [26], Mealer et al. [27], and Hinderer et al. [28] and are essential for sustaining nurse well‐being and quality patient care.

### 4.1 Study Limitations

This study used a convenience sampling method across three tertiary hospitals within HMC, potentially limiting the generalizability of findings to other HMC facilities or broader healthcare contexts. The cross‐sectional design captures data at a single time point, restricting the ability to assess trends over time.

The focus on clinical nurses excluded perspectives from newly licensed nurses, advanced practitioners, and nurse leaders, who may experience different levels of compassion satisfaction and fatigue. Reliance on self‐reported measures may have introduced response or social desirability bias. In addition, although the ProQOL is widely used internationally, the questionnaire was not formally validated in the current study population/context, which may affect measurement accuracy and cultural applicability of the scores. Furthermore, this study did not include key organizational variables—such as workload, shift length, staffing ratios, leadership support, or unit‐level stressors—which are known to influence compassion satisfaction and fatigue. These limitations should be considered when interpreting the findings and highlight the need for future research incorporating longitudinal designs, objective indicators, and organizational‐level factors.

### 4.2 Implication to Nursing Practice

Compassion is fundamental to nursing and critical to both patient care quality and nurse well‐being. This study highlights that while most nurses report moderate compassion satisfaction, a significant number experience low satisfaction and moderate fatigue, potentially impacting care and job performance.

Implementing evidence‐based self‐care strategies—such as mindfulness, reflective selftalk, breathing techniques, gratitude practices at workplace and sleep hygiene—can enhance emotional resilience. Integrating these concepts into nursing education and professional development is essential for long‐term emotional sustainability.

In the educational context, integrating concepts related to compassion fatigue, resilience, and emotional self‐awareness into nursing curricula may influence how nurses understand and manage the emotional demands of clinical practice over time.

## 5 Recommendation

Future research should explore the intricate relationships between fatigue, job satisfaction, employee engagement, staff retention, and coping strategies among clinical healthcare professionals. There is a compelling need to design and assess resilience training programs aimed at mitigating the effects of these factors. Furthermore, examining the role of organizational culture, leadership support, and team dynamics in building resilience is essential. Research should also focus on the significance of work‐life balance, mental health interventions, and technological innovations in alleviating burnout. Additionally, the impact of these factors on patient outcomes, such as quality of care and patient satisfaction, should be a central focus for future studies.

## 6 Conclusion

Compassion fatigue poses a significant challenge for nurses, resulting from the physical, mental, and emotional demands of providing empathetic care, especially in high‐acuity settings. Understanding and applying the principles of ProQOL can positively influence both the work environment and patient care outcomes. This study contributes to the growing body of knowledge on compassion fatigue and satisfaction among clinical nurses in a tertiary care context. Importantly, subgroup differences identified in this study should be interpreted as exploratory and descriptive rather than causal.

By applying the Professional Quality of Life (ProQOL) framework, healthcare organizations can better identify areas for intervention aimed at enhancing nurse well‐being. Strategies focused on resilience, self‐care, professional development, and supportive organizational environments may help mitigate burnout and STS while fostering compassion satisfaction. A holistic and evidence‐informed approach is essential to promote nurse retention, optimize performance, and ensure sustainable, high‐quality patient care.

## Author Contributions

S.K.P., R.R., and B.A. conceived and designed the study. Data collection was carried out by S.K.P. and K.P., while data analysis and interpretation were performed by S.K.P. and K.S. The study was supervised by R.R., K.S., and B.A. The manuscript was written by S.K.P., R.R., B.A., K.S., and K.P., with critical revisions for important intellectual content provided by S.K.P. and K.S. All authors reviewed and approved the final version of the manuscript.

## Conflicts of Interest

The authors declare no conflicts of interest.

## Transparency Statement

The lead author, Surekha Kiran Patil, affirms that this manuscript is an honest, accurate, and transparent account of the study being reported; that no important aspects of the study have been omitted; and that any discrepancies from the study as planned (and, if relevant, registered) have been explained.

## Data Availability

The data that support the findings of this study are available from the corresponding author upon reasonable request.
